# GBAF, a small BAF sub-complex with big implications: a systematic review

**DOI:** 10.1186/s13072-020-00370-8

**Published:** 2020-11-03

**Authors:** Sarah M. Innis, Birgit Cabot

**Affiliations:** Department of Animal Sciences, Purdue University, West Lafayette, IN USA

**Keywords:** Chromatin remodeling, Epigenetic modification, Gene expression, Pluripotency, Embryonic development, Neurodevelopment, Oncogene, Tumor suppressor

## Abstract

ATP-dependent chromatin remodeling by histone-modifying enzymes and chromatin remodeling complexes is crucial for maintaining chromatin organization and facilitating gene transcription. In the SWI/SNF family of ATP-dependent chromatin remodelers, distinct complexes such as BAF, PBAF, GBAF, esBAF and npBAF/nBAF are of particular interest regarding their implications in cellular differentiation and development, as well as in various diseases. The recently identified BAF subcomplex GBAF is no exception to this, and information is emerging linking this complex and its components to crucial events in mammalian development. Furthermore, given the essential nature of many of its subunits in maintaining effective chromatin remodeling function, it comes as no surprise that aberrant expression of GBAF complex components is associated with disease development, including neurodevelopmental disorders and numerous malignancies. It becomes clear that building upon our knowledge of GBAF and BAF complex function will be essential for advancements in both mammalian reproductive applications and the development of more effective therapeutic interventions and strategies. Here, we review the roles of the SWI/SNF chromatin remodeling subcomplex GBAF and its subunits in mammalian development and disease.

## Background

**Fig. 1:**
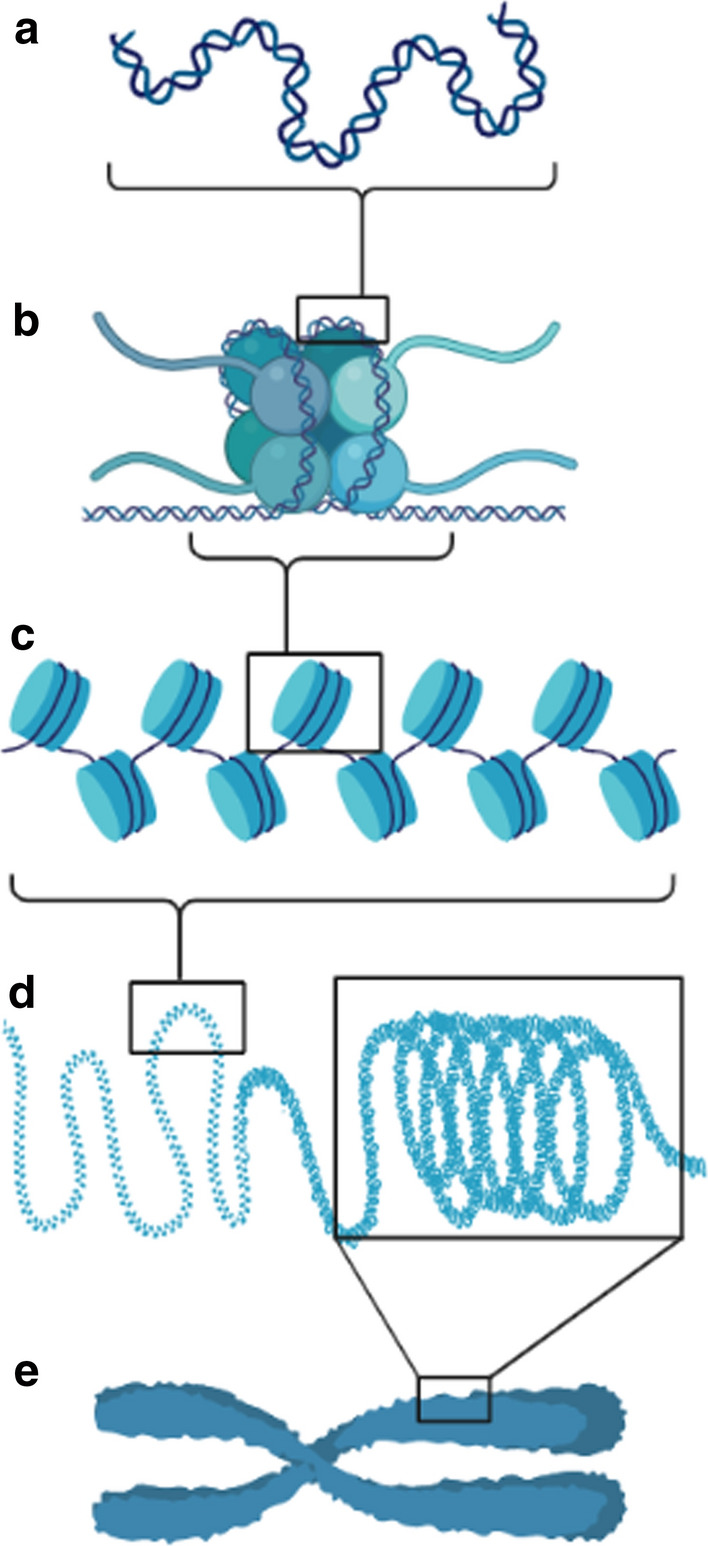
Schematic representation of chromatin arrangement. **a** Double stranded DNA (dsDNA) forms the genetic material. **b** dsDNA wraps around a histone octamer, forming a nucleosome. **c** Nucleosomes form the repeating organizational units of chromatin. **d** Condensation of looped chromatin forms chromatin fibers. **e** Further compaction of chromatin fibers forms chromatids and, ultimately, chromosomes. Figure created using BioRender.com

The dynamic modification of chromatin architecture plays a critical organizational role in the eukaryotic genome. Chromatin consists of fundamental repeating units called nucleosomes, in which DNA is wrapped tightly around a histone octamer [1], allowing for efficient genome compaction into the nucleus (see Fig. 1). This functional organization not only achieves the feat of genomic condensation, but it also enables the epigenetic regulation of the genome to modulate gene expression. Coupled with the necessity of maintaining dynamic organization, regulating chromatin accessibility to transcriptional machinery presents an interesting challenge for these epigenetic mechanisms.

The post-translational modification (PTM) of histones, such as methylation and acetylation, has been shown to recruit protein complexes and molecular machinery to these modification sites to initiate enzymatically driven structural reorganization [2]. As a result, this process of PTM followed by protein complex recruitment regulates not only gene expression, but also DNA replication and repair [3]. Essential to these processes are ATP-dependent chromatin remodeling complexes, which play a central role in regulating both chromatin architecture and gene expression via their interactions with nucleosomes [4]. Multiple families of these complexes exist, most notably SWI/SNF, ISWI, CHD, and INO80, all of which are characterized by their central ATPases and associated subunits [5]. As such, distinct complexes are formed with varying complexities and combinatorial assemblies involved in directing cell differentiation and lineage-specific transcription [6]. In addition to this, the variety of subunit combinatorial assemblies found across these ATP-dependent remodeling complexes is reflective of the increased evolutionary and biological complexity of the organisms in which they are found. Therefore, particularly in mammalian species, it is not surprising that mutations in the many genes encoding complex subunits have known associations with cancers and numerous developmental disorders, reinforcing the importance of continuing to investigate the myriad mechanistic roles these complexes play. Here, we review the GLTSCR1/like-containing BAF complex (GBAF), a subcomplex of the ATP-dependent mammalian SWI/SNF (switch/sucrose nonfermenting) family, focusing specifically on the roles of GBAF and its subunits in development and disease.

## Overview of SWI/SNF family chromatin remodeling complexes

### Evolutionary diversification of SWI/SNF complexes

SWI/SNF chromatin remodeling complex function is highly conserved in multiple eukaryotic species, including *Saccharomyces cerevisiae, Drosophila melanogaster,* and *Homo sapiens*. The first set of genes belonging to SWI/SNF were identified and described in *S. cerevisiae* following two genetic screens studying altered gene expression in mating-type switching and sucrose fermentation. In one screen, *SWI* genes were shown to play a critical role in maintaining yeast mating-type switching via expression of the *HO* gene [7]. In another screen, *SNF* genes were shown to contribute to the expression of *SUC2*, an essential gene involved in supporting yeast survival on different carbon nutrient sources [8, 9]. Following the supposition that multiprotein complexes were involved in regulating transcriptional activation and repression [10], the first link between SWI/SNF and chromatin remodeling was determined by identifying SWI/SNF mutation suppressors in yeast, specifically in genes encoding chromatin components such as histones [11–13]. Further research in yeast has identified that SWI/SNF is principally concerned with transcriptional activation rather than repression, which is not altogether unexpected given the high percentage of active genes in this organism [14, 15]. In yeast, the ~ 1.15 MDa SWI/SNF complex is made up of around 11 total polypeptide subunits, including a conserved catalytic core comprising 3 polypeptides (the central ATPase Swi2/Snf2, Swi3, Snf5) [16, 17].

In *Drosophila*, the 2-MDa BAP (Brahma-associated-proteins) SWI/SNF complex contains a central ATPase corresponding to Swi2/Snf2 in yeast called Brahma (BRM), the core subunit from which this complex derives its name. BRM was originally identified to be a dominant suppressor of Polycomb complexes, which are involved in developmental differentiation and transcriptional repression [18, 19]. In addition to BRM, BAP contains multiple subunits also conserved in yeast and humans, including BAP155 (Swi3 in *S. cerevisiae* and BAF155 in *Homo sapiens*), BAP45 (Snf5 in *S. cerevisiae* and BAF47/SNF5 in *H. sapiens)*, and actin-associated proteins BAP55/BAP47 (Arp7/9 in *S. cerevisiae* and BAF53a/b in *H. sapiens*) [20]*.* Of note, the primary targets for BAP do not appear to be histones, as revertant mutations have not been discovered in genes encoding histones, unlike in yeast. Instead, BAP seems to function in opposition to Polycomb complexes, though research into BAP function is ongoing [21, 22].

The initial identification of the 2 MDa BAF (BRM/BRG1 Associated Factors) SWI/SNF subcomplex in mammals began with the discovery of hBRM and BRG1, homologs of Swi2/Snf2 in yeast [23]. In addition to the subunits shared with both *S. cerevisiae* and *Drosophila* (BAF155, BAF47, and BAF53a/b), BAF complexes typically contain several additional subunits conserved with yeast, though there has been significant evolutionary divergence from yeast to mammalian SWI/SNF. These subunits include BAF60 (Swp73 in yeast), BAF45b/c/d (Swp82), and BAF250a/b (Swi1) [24]. Additionally, BAF contains BAF57, SS18, B-cell CLL/lymphoma 7 (BCL7) protein family members a/b/c, BCL11, and actin, for a total of 10–15 subunits encoded by at least 29 genes belonging to around 15 different gene families [22, 25–27].

As a point of consideration, while a fully assembled BAF complex generally contains at least 10 subunits, a minimum catalytic core consisting of BRG1, BAF47/SNF5, BAF155, and BAF170 is able to achieve in vitro nucleosome remodeling activity comparable to the entire complex [26, reviewed by 28]. In fact, under in vitro conditions, BRG1/BRM alone can remodel nucleosome templates to some extent, but the remodeling efficiency compared to the fully assembled complex is greatly reduced [26]. Recent work has explored whether the minimum catalytic core exists in vivo, but no physiological evidence was recovered suggesting such a tetramer exists in mammalian cells [29].

The larger, but less abundant, polybromo-BAF (PBAF) complex is distinguishable from BAF due to its incorporation of four unique subunits, ARID2, PBRM1, BAF45d, and BRD7 [26]. More recently, another mammalian BAF complex was identified and coined GBAF (also called non-canonical or ncBAF), distinguishable from the other BAF complexes by the exclusion of several canonical BAF subunits (ARID1A/1B/2, BAF45, BAF47, and BAF57), as well as the inclusion of two unique subunits, bromodomain-containing 9 (BRD9) and the mutually exclusive paralogs glioma tumor suppressor candidate region gene 1 (GLTSCTR1) and glioma tumor suppressor candidate region gene 1-like (GLTSCR1L) [27]. Table 1 provides a comparison of GBAF subunit homology across species. Most GBAF subunits have homologs in yeast and drosophila, with the exceptions of GLTSCR1/L, BRD9, and SS18.

**Table 1:** GBAF subunits and their species-specific homologs in mammalian development and disease

Gene	*Saccharomyces cerevisiae*	*Drosophila melanogaster*	Mammalian homolog	Developmental roles	Disease implications
ACTL6A	Arp4, 7, 9	BAF55	BAF53a	Hematopoietic development [72]; neuronal develoment [75]; mouse ESC survival [195]; regulation of ESC differentiation into primitive endoderm [196]	Cancer [159–161, 163–165]
BICRA^a^			GLTSCR1	Mouse ESC survival [27]	Cancer [27, 96–100, 103, 105–107]
BICRAL^a^			GLTSCR1		Cancer [27]
BRD9^a^			BRD9	Naïve ESC pluripotency [55]	Cancer [122, 123, 126–128, 130]
SMARCA4	Swi2/Snf2	BRM	BRG1	Zygotic genome activation [59, 60]; loss is perimplantation lethal [60, 61]; ESC differentiation [64]; hematopoietic development [69–71]; neuronal development [75]; neural tube closure [197]	Coffin–Siris syndrome [78, 82, 83]; cancer [60, 138, 140–145]
SMARCC1	Swi3/Snf12	BAP155/MOR	BAF155	*Nanog* silencing [55]; ESC differentiation [64]; loss is perimplation lethal [64]; neural tube closure [197]	Autism spectrum disorder [79]; Cancer [148–152, 156–158]
SMARCD1	Swp73/Snf12	BAP60	BAF60a		Neurodevelopmental disabilities [84]; Cancer [170, 172, 174, 175, 177]
SS18^a^			SS18	Knockdown and replacemet with CREST promotes neural developmental progression [76]	Cancer [107, 182, 185–188, 190, 191]

Not all subunits may have a defined homolog across species. References listed are from the prevailing literature and do not represent an exhaustive list for each category

^a^ Indicates a unique mammalian gene with no known homologs found in yeast or fly

**Fig. 2:**
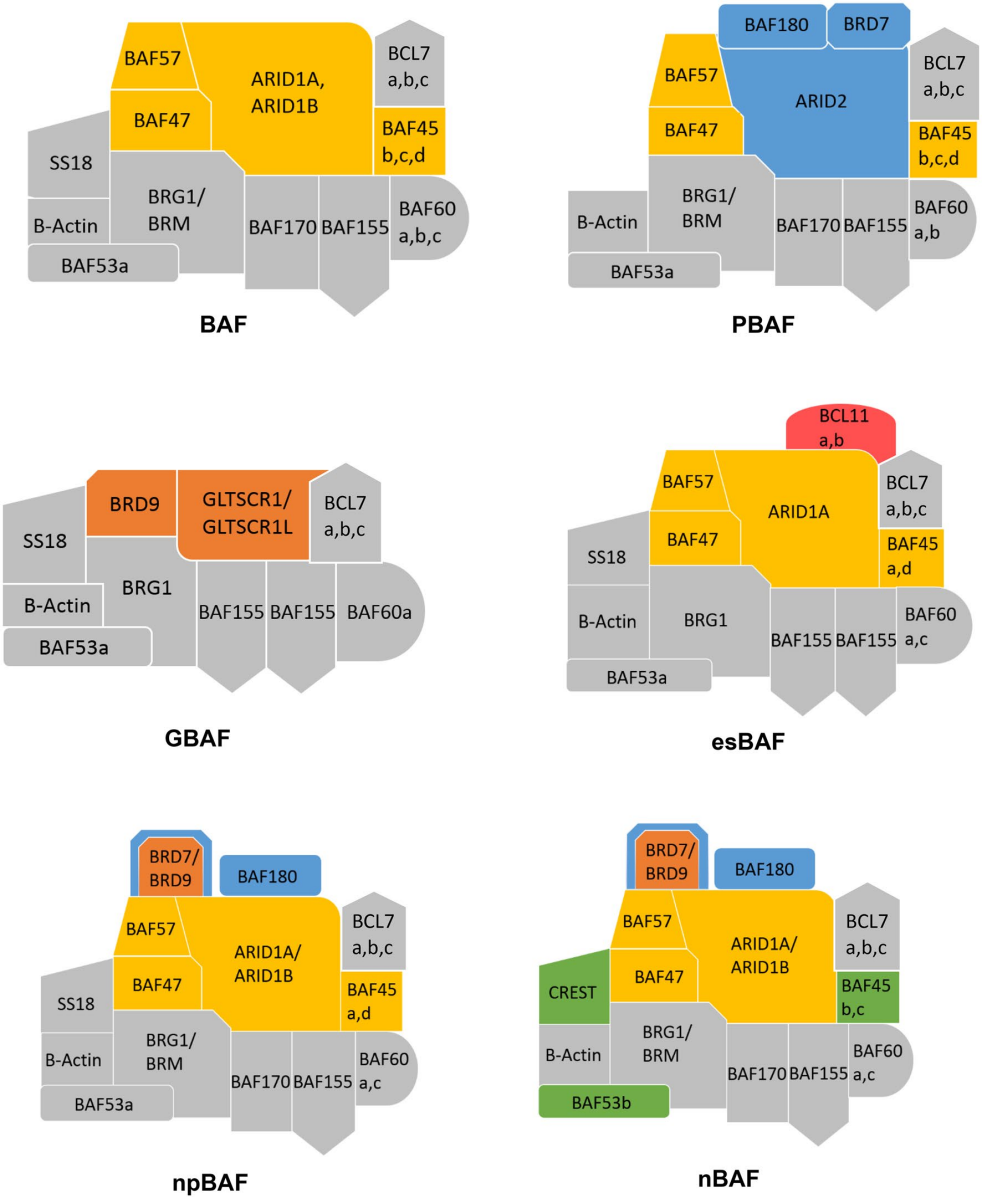
Schematic representations of the BAF, PBAF, GBAF, esBAF, npBAF, and nBAF complexes. Gray subunits are shared across multiple complexes. Gray subunits are general BAF-subcomplex components, yellow subunits are BAF-specific, blue subunits are PBAF-specific, orange subunits are GBAF-specific, the red subunit is esBAF-specific, and the green subunits are nBAF-specific following npBAF/nBAF switching. Subunits with two colors indicate that a subunit could be a defining component from either represented complex

In summary, mammalian BAF complexes are affiliated with both transcriptional activation and repression, further highlighting the evolutionary diversification of these organism- and lineage-specific complex assemblies [29]. An overview of different BAF-subcomplex assemblies is provided in Fig. 2.

### Mechanistic overview of ATP-dependent chromatin remodeling

Key components of ATP-dependent chromatin remodeling are histone-modifying enzymes, which coordinate with remodeling complexes to facilitate gene activation and repression [20, 30–32]. These enzymes are responsible for recognizing and generating covalent marks on histone tails, including (among others) methylation, acetylation, and phosphorylation [33]. These marks are recognized by ATP-dependent chromatin remodelers, resulting in nucleosome unwrapping, mobilization, ejection, or exchange via ATP-hydrolysis [20, 34, 35]. Nucleosome alterations result in a chromatin architecture state conferring either transcriptional activation or repression at these sites, depending upon the positioning of the nucleosomes. A central mechanism for ATP-dependent remodelers has been proposed, in which ATP-dependent translocation results in the propulsion of DNA along the surface of a histone [36]. Sirinakis and coworkers measured the speed of these DNA translocation events to be about 25 base pairs per second in the ATP-dependent RSC remodeling complex in yeast [37].

Recent studies into yeast and human SWI/SNF complexes have utilized cryo-electron microscopy (EM) to visualize structural characteristics and postulate mechanistic features of these complexes. In yeast RSC, five protein modules have been identified, each contributing to remodeling complex function and the formation of transcriptionally active promoter regions in a nucleosome-free state. Similarly, three protein modules have been found in nucleosome-bound yeast RSC and human BAF using cryo-EM [38–42].

In addition to the differences in module/lobe number between nucleosome-bound and nucleosome-free RSC, Wang et al. identified a key positional difference between the two states. While both contain positional overlap in their respective base modules, the actin-related protein (ARP) module of nucleosome-bound RSC is rotated away from the nucleosome-binding lobe to allow for nucleosome occupation [40]. Furthermore, the authors showed that when compared to human BAF, yeast RSC displays conservation of structural features and function of distinct subunits, despite the complex-specific subunit organization between species. Structural determination of nucleosome-bound RSC and BAF has also distinguished some assembly homology between RSC, BAF, and PBAF [41], though nucleosome binding sites relative to modules were found to be distinct between these complexes [42]. Taken together, these studies serve as a valuable contribution towards a greater understanding of SWI/SNF complex function, structure and mechanisms of action, all of which are essential components of studying these complexes within development and disease contexts.

The ability of the BAF catalytic core subunits to perform chromatin remodeling in vitro [43], as well as discoveries identifying BAF complex function in cellular differentiation/conversion [44, 45] and human diseases such as cancer [46], indicate that these ATP-dependent remodelers affect a variety of different physiological conditions and pathological processes [22]. Consistent with this hypothesis are observations in mammalian development, wherein chromatin remodeling structures can respond to different stimuli such as stress, extracellular signals, or developmental cues in a context-specific manner [47–49]. Details about some of these additional roles and implications will be discussed in later sections, but with the expanding knowledge base surrounding SWI/SNF in development and disease, the pursuit of research in these areas is sure to continue garnering interest.

## GBAF and its subunits in mammalian development

### Regulation of pluripotency

For embryonic stem cells (ESCs) and induced pluripotent stem cells, pluripotency and self-renewal are defining characteristics. Derived from the inner cell mass (ICM) of mammalian blastocysts, ESCs can be cultured indefinitely and serve as the basis for the three embryonic germ layers and germ cells [50–52]. Pluripotency in ESCs is partly conferred by the expression of transcription factors (TFs) such as SOX2, OCT4, and homeobox protein NANOG, which have a primary function in supporting self-renewal and suppressing lineage-specific genes [29, 52, 53]. In mouse ESCs, a specialized BAF complex called esBAF (for ESC BAF) is characterized by the presence of several dedicated BAF complex subunits, including BRG1, BAF155, and BAF60a [54]. Uniquely, esBAF cooperates with the master pluripotency regulators OCT4, SOX2, and NANOG to regulate ESC-specific gene expression and repress differentiation [54, 55]. This interaction between key pluripotency regulators and esBAF provides a compelling link between BAF complex function and pluripotency maintenance requirements.

To that end, multiple defined pluripotency regulators are known to interact with BAF complex subunits [54, 56, 57], and recent research has identified an increasing number of links between GBAF subunit function and pluripotency maintenance, indicating GBAF regulation of the ESC transcriptome [55].

For example, BRD9 has been shown to maintain naïve pluripotency in embryonic stem cells by regulating pluripotency factors such as NANOG, ultimately preventing developmental transition to a primed epiblast state [55]. Interruption of BRD9 transcription resulted in decreased ESC growth. In the same study, it was determined that GBAF is recruited to chromatin via bromodomain-containing 4 (BRD4), which is recognized by the bromodomain of BRD9 in its acetylated form, highlighting an interesting functional similarity between GBAF and esBAF [55].

Gatchalian et al. also provided a compelling comparison of the genomic binding character between GBAF and esBAF. BRD9 binding was more enriched at promoter regions, whereas ARID1A was more enriched at enhancers. Furthermore, while the general pluripotency regulators OCT4, SOX2, and NANOG showed greater occupancy at esBAF sites in TF association analyses, CCCTC-binding factor (CTCF) displayed stronger association with GBAF sites. Given the distinct TF associations between different BAF-subcomplex assemblies, Gatchalian et al. ultimately postulate the likelihood that cooperation must exist between GBAF and esBAF to ensure successful naïve pluripotency maintenance [55].

In contrast to BRD9, genetic deletion of GLTSCR1 has not been shown to adversely affect mouse ESCs [27]. To be sure, the insights into the essential nature of GBAF and its dedicated subunit BRD9 in modulating naïve pluripotency will serve as a cogent basis for future work into mammalian developmental requirements.

### Roles in embryonic development

Given its roles in regulating aspects of the pluripotency transcriptional network, it follows that GBAF and its subunits would also be affiliated with both embryonic development and regulation of critical developmental milestones. In mouse ESCs, both GLTSCR1 and its paralog GLTSCR1L are expressed, but upon retinoic acid-induced differentiation, *GLTSCR1* is upregulated while *GLTSCR1L* is downregulated [55]. This may suggest that GLTSCR1 is the more dominant GBAF paralog, but it also indicates that different subunit composition preferences may exist depending on the context of specific developmental stages and requirements.

Recent research by the authors has identified the presence of BRD9 and GLTSCR1 in porcine oocytes and cleavage stage embryos, as well as in porcine trophectoderm and fetal fibroblast cells [58]. While study into the significance of BRD9 and GLTSCR1 in mammalian development is still in its early stages, the associations of other GBAF and SWI/SNF subunits with different aspects of development will provide, at the very least, a sound basis for continuation of research in this area.

The following subunits are shared between different BAF subcomplexes and not exclusive to GBAF; however, it is worth addressing their importance in embryonic development. The BAF complex central ATPase BRG1 is a critical component in regulating mammalian embryonic development. Multiple studies have discussed the necessity of BRG1 at the pre- and peri-implantation stages in mice and pigs, as loss of BRG1 is lethal for embryos [59–61]. The contributions of BRG1 in early embryonic development also include zygotic genome activation (ZGA), a crucial developmental checkpoint responsible for establishing a gene expression profile necessary for further differentiation [62, 63]. BRG1 and BAF155 show widespread association overlap in the ESC genome, supporting the role these subunits have in chromatin remodeling during this developmental stage [54]. Panamarova et al. showed that in mouse ESCs, the BAF155 scaffolding protein regulates in vivo BAF complex assembly, as well as lineage specification in blastocysts. BAF155 loss results in peri-implantation lethality, while *Baf155* overexpression is also detrimental, resulting in developmental arrest observed at the blastocyst stage [64]. In porcine embryos, BAF155 is highly expressed in early cleavage-stage embryos, with decreased expression at blastocyst-stage differentiation in vitro [65]; this reflects similar results seen in murine embryos [64, reviewed by 66].

### Roles in cardiac development and hematopoiesis

Recent research has identified a role for BRD9 in regulatory T-cell (Treg) development via control of Foxp3 [67], a TF known to coordinate differentiation and functional events in Tregs [68]. Loo et al. showed that GBAF promoted the expression of *Foxp3*, an observation in contrast to the repressive effects on *Fox3p* by PBAF. Furthermore, the authors demonstrated that BRD9 chemical degradation or ablation of *Brd9* compromised Treg function in both in vitro and in vivo conditions [67]. These results are especially relevant to the search for therapeutic targets for immune system control in autoimmune diseases and cancers; the links between T-cell function and GBAF suggest a potentially viable target in this pursuit.

Non-exclusive GBAF subunits have also been shown to have significant roles in hematopoiesis. BRG1 is essential for immature T-cell development [69], including mediating glycoprotein lineage specification [70]. During granulocyte development, the progression of granulocyte-colony stimulating factor (G-CSF) is blocked by dominant negative mutant BRG1 expression [71], solidifying the importance of this subunit in immune cell development. Conditional deletion of *BAF53a* caused multilineage failure, aplastic anemia, and rapid lethality in human bone marrow cells in an environmentally independent fashion [72].

### Roles in neurodevelopment

During nervous system development in vertebrates, division of neural progenitors is followed by mitotic exit of progeny and migration of these progeny to specific sites, leading to the determination of their physiological fate [73, 74]. At mitotic exit, a microRNA-mediated switch occurs in the ATP-dependent chromatin remodeling complex assembly from npBAF (neural progenitor BAF) to nBAF (for neuronal BAF), resulting in complete conversion of neural progenitors to neurons [44]. Particularly in npBAF, multiple subunits are shared with GBAF (BRG1, BAF155, BAF60a, BAF53a, SS18), though several of these subunits are switched to a combinatorial assembly specific to nBAF upon precisely controlled mitotic exit (SS18 to CREST, BAF53a to BAF53b, BAF45a/d to BAF 45 b/c, and differential expression of BAF155/170) [75, 76]. For a schematic, see Fig. 2.

In general, study into the significance of GBAF-exclusive subunits BRD9 and GLTSCR1 in mammalian development is still in its early stages, and as a result, data are somewhat limited. With this in mind, the associations of other non-exclusive GBAF and SWI/SNF subunits may not allow for specific, precise conclusions to be made about GBAF and its possible roles in development. However, inclusion of these subunits in this review will help to create a comprehensive understanding of the presumptive implications GBAF has during key developmental processes and disease pathologies. Certainly, research into the significance of GBAF and mammalian development will continue to encounter intersections with the implications of this BAF subcomplex and its subunits in disease development, especially as more information providing further mechanistic clues continues to be uncovered. An overview of nomenclature and GBAF subunit roles in development and disease is provided in Table 1.

## GBAF and its subunits in mammalian disease

### Roles in neurodevelopment disorders

The proper functioning of BAF complexes in mammalian development is critical for establishing cell fates and ensuring proper developmental checkpoints are reached. It is not surprising that mutations in genes encoding BAF subunits are associated with a wide variety of diseases and disorders known to affect organisms across many different life stages [77]. With defined contributions toward neural development, it is becoming increasingly clear that interruptions in GBAF function and its subunit composition may have the capacity to engender disease development and progression in neural systems. However, while some subunits shared across other BAF subcomplexes have defined roles in neurodevelopment disorders, specific contributions for BRD9, GLTSCR1/L, and GBAF have yet to be discovered. A brief overview of some of the defined roles in non-exclusive GBAF subunits is briefly included here to provide a basis for understanding the known associations between mammalian BAFs and atypical neurodevelopmental function.

The implications of BRG1 and BAF155 mutations regarding neurodevelopment disorders are of great significance. Partial deletion and missense mutations in *BRG1* and *BAF155* are associated with the development of Coffin–Siris syndrome (CSS) and autism spectrum disorder (ASD) in humans [78, 79]. CSS is a rare congenital syndrome characterized by coarse facial features, growth deficiency, intellectual disability, hypoplasia of the fifth distal finger and fingernails, feeding difficulties, hypertrichosis, and frequent infections [80, 81]. Out of 180 cases of CSS reported across several studies, around 110 of these included a mutation in BAF complex genes, including *BRG1* [81–83].

Several other GBAF-associated subunits have known links to neurodevelopment disorders. For example, in three unrelated human subjects presenting with varying degrees of development disabilities, heterozygous variants in *BAF53a* (*ACTL6a*) were identified, suggesting a previously unknown link between this subunit and neurodevelopmental delays [84]. De novo mutations in *BAF60a* were identified in 4 out of 5 individuals presenting with developmental delay, intellectual disability, hypotonia, feeding difficulties, and small hands and feet [85].

To sum, given the essential roles many of the same subunits play in neurodevelopment, it is altogether unsurprising that aberrant GBAF subunit expression would have detrimental effects on cognition and contribute to neurodevelopmental defects. As more research is done to further elucidate mechanistic links and relationships between SWI/SNF subunits and neurodevelopment disorders, it is likely that BAF/GBAF subunits will remain at the forefront of study. Still, gaps in our knowledge are evident, and much new insight is required to better understand both the mechanistic contributions of chromatin remodeling complexes and any therapeutic potential of targeting them.

### Roles in cancer: tumor suppressors and oncogenes

Genes encoding chromatin remodeling proteins are among the most frequently mutated in cancer, with over 20% of cancers containing mutations in genes coding for SWI/SNF subunits [25, 86, 87]. SWI/SNF mutation rates are as high as 75% in ovarian clear cell carcinoma and 57% in clear cell renal carcinoma [86], and the number of known associations of SWI/SNF subunit mutations with other cancer types continues to increase. Given their roles in cancer development when mutated, it follows that multiple SWI/SNF subunits have been shown to have roles in tumor suppression [87, 88]. Early studies into the potential involvement of SWI/SNF and tumor suppression were spurred by observations that core BAF subunits were absent in immortalized cell lines [89], and subsequent research found that the BAF core subunit BAF47 (SNF5) was disabled in almost all cases of malignant rhabdoid tumors (MRTs) [90, 91], an aggressive childhood cancer. Later, the tumor suppressor role was confirmed in mouse models [92–95] via conditional inactivation of *Snf5*, wherein around 15–30% of *Snf5* heterozygous mice developed sarcomas similar to human MRTs, and biallelic inactivation of *Snf5* caused fully penetrant T cell lymphoma to develop at a surprisingly rapid median onset of 11 weeks [95].

**Fig. 3:**
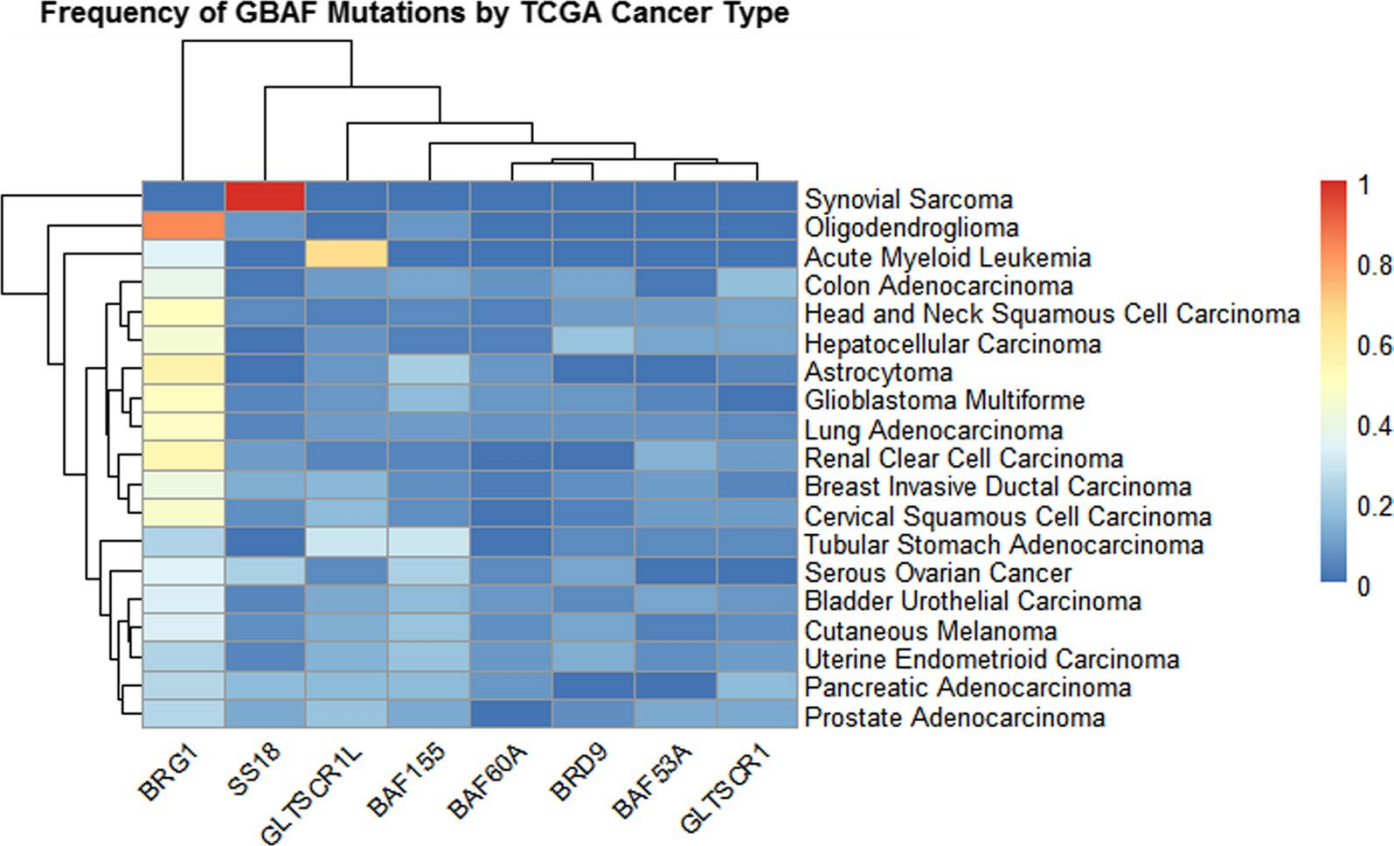
Heatmap representing the frequency of mutated GBAF subunits in each TCGA cancer type. Color indicates frequency of mutated subunit in each cancer type. Study data provided by [193, 194] are represented. Data obtained from cBioPortal

**Fig. 4:**
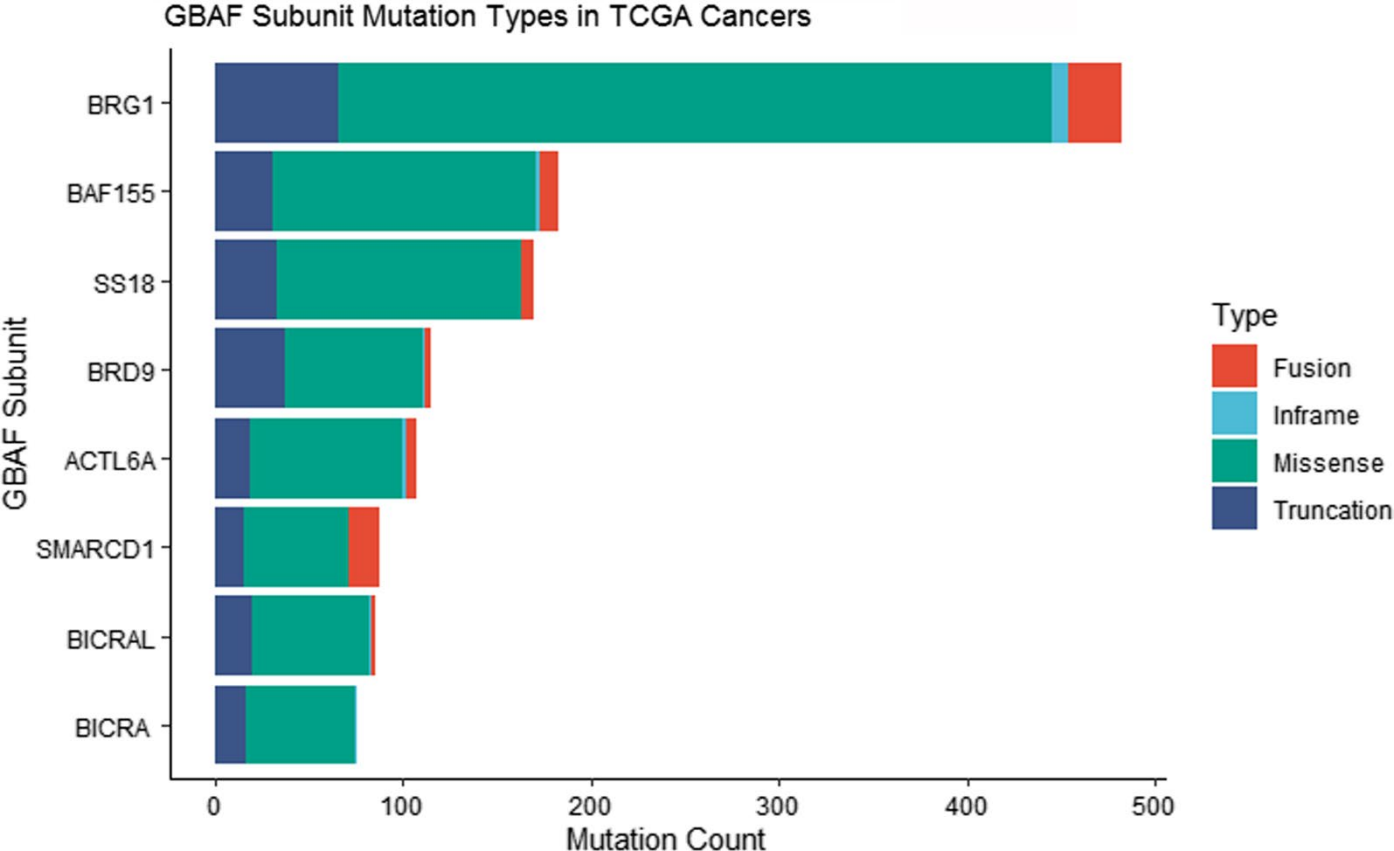
Stacked bar chart showing the number of GBAF subunit mutations across The Cancer Genome Atlas (TCGA) study data obtained from cBioPortal. Study data provided by [193, 194] are represented. Color indicates mutation type

The following sections will profile GBAF subunits and provide an overview of known oncogenic and tumor-suppressive associations, as well as potential therapeutic approaches being considered based on the prevailing literature. Particular emphasis is placed on GBAF-specific subunits BRD9 and GLTSCR1. Figure 3 shows the frequency of GBAF subunit mutations in different cancer types. The distribution of various mutations found in GBAF subunits is presented in Fig. 4.

### GLTSCR1/GLTSCR1L

Since *GLTSCR1* was first identified as a potential tumor suppressor gene in human gliomas [96] nearly 20 years ago, several associations have been uncovered between this GBAF subunit and cancer. A few years after this initial discovery, mutations in *GLTSCR1* were shown to be associated with oligodendroglioma development and progression, as well as glioma survival [97]. The authors of this study postulated that due to observations of *GLTSCR1*-exon polymorphism phenotypes, chromosome arm alterations might not be the only indicator of survival in glioma cases [97]. Subsequent research identified a *GLTSCR1* polymorphism corresponding to a 3.5-fold increased risk of developing meningioma [98], further implicating *GLTSCR1* with cancer types displaying rapid fatality [99, 100].

In addition to brain tumors, recent work has elucidated a wider range of cancer associations with GLTSCR1. After it was found that several loci associated with prostate cancer aggressiveness were located on chromosome 19 [101, 102], the same chromosome of interest in the aforementioned brain tumor studies, the presence of *GLTSCR1* on this chromosome was an indicator for a potential link between prostate cancer and GLTSCR1 [103]. Indeed, the same study revealed that the expression of GLTSCR1 protein had a striking association with advanced clinical stage, enhanced tumor invasion, and lymph node and distant metastasis in prostate cancer tissue samples. Interestingly, the authors noted that prostate cancer patients with high expression levels of *GLTSCR1* had a significantly shorter life expectancy when compared to prostate cancer patients with lower expression [103]. Knockout of *GLTSCR1* in metastatic prostate cancer cell line PC3 resulted in a noticeable decrease in both colony formation and proliferation in this cell type, and similar effects were observed following *GLTSCR1L* knockout, suggesting a dependency of PC3 cells on GLTSCR1 and GLTSCR1L [27]. In general, much less information exists linking GLTSCR1L to cancer compared to other GBAF subunits. However, the putative observation that GLTSCR1L associates with and mediates enhancer transcription [104] combined with the results seen by Alpsoy and Dykhuizen in PC3 cells provides a plausible basis for future research with this GBAF subunit.

Investigation of the relationship between gene polymorphisms and lung cancer risk in the Chinese population revealed single nucleotide polymorphism (SNP) haplotypes had a joint interaction effect with smoking, including multiple *GLTSCR1* SNPs [105]. Another study demonstrated that wild-type GLTSCR1 was able to bind to BRD4, resulting in reduced expression of target genes (such as the oncogenes SLC2A1 and SLC2A3) and inhibition of colorectal cancer (CRC) metastasis [106]. The authors detailed the discovery that a microsatellite site in *GLTSCR1* was responsible for a frameshift mutation causing a truncated GLTSCR1 protein to be produced. This protein product was incapable of nuclear import and BRD4 interaction in a CRC line displaying high microsatellite instability [106]. GLTSCR1 has also been shown to be required for synovial sarcoma cell survival [107]. Walker and coworkers showed a potential connection between GLTSCR1 and AML, but identification of a mechanistic link between this subunit and disease is still needed [108].

### BRD9

BRD9 belongs to a family of bromodomain (BRD)-containing proteins that recognize *N*-acetylated lysine molecules on proteins such as histones. This recognition allows for the modulation of cellular functions like chromatin remodeling and gene transcription. The discovery that several BRD-containing proteins are involved in inflammation [109, 110] and tumorigenesis [111] has made them a compelling target for small-molecule inhibition studies [112]. One of the first selective inhibition studies focused on BRD4 [113], a BRD9 paralog and BRD-containing protein family member with epigenetic mediatory activities and known roles in cancers, including chronic lymphocytic leukemia [114] and human squamous carcinoma [115]. Recent research found that BRD7, another BRD9 paralog, had several oncogenic links [116–118]. The bromodomains of BRD7 and BRD9 have around a 72% residue similarity [119], and therefore, many of the small-molecule inhibitors targeting the BRD9 bromodomain will also inhibit BRD7 [119, 120]. The simultaneous inhibition of BRD7 and BRD9 presents obvious challenges to researchers hoping to reduce the risk of pleiotropic effects. Recently, compounds providing selective inhibition of BRD9 over BRD7 have been identified [120, 121]. As more compounds are developed which offer greater selectivity for specific subunit targeting, the potential for uncovering more intricate details about the roles distinct subunits have in development and disease is sure to increase.

In contrast to GLTSCR1, a definite mechanistic link between AML and BRD9 has been identified. In AML cell lines, it was shown that the bromodomain of BRD9 was responsible for promotion of MYC expression and proliferation of AML cells [122]. Interestingly, this study also determined that the BRD9 bromodomain could be replaced with different bromodomains without interrupting AML cell maintenance, which allowed the authors to explore several novel small-molecule inhibitors for BRD9 that were capable of selective disruption of AML cell proliferation [122]. In squamous cell lung cancer (SqCLC), proliferative control and tumorigenesis appears to be managed by at least one tumor-suppressive miRNA via BRD9. Huang et al. showed that Mi-R140-3p is downregulated in SqCLC, whereas BRD9 expression is upregulated. High BRD9 levels in SqCLC patients were correlated with poor prognosis. Conversely, inhibition of BRD9 had a negative impact on tumorigenesis, due in part to BRD9’s influence on the down-regulation of c-myc [123]. In cancers like AML and SqCLC, the apparent dependence of these cancer types on BRD9 may be a viable avenue for investigations into disease progression and treatment.

More evidence suggests that SWI/SNF complexes themselves, as well as their constituents, are promising potential targets for therapeutic approaches in certain cancers. Such an example can be found in two cancer types which share a BAF complex perturbation of BAF47 (SNF5), MRT and synovial sarcoma (SS). MRTs are aggressive, highly malignant renal tumors which primarily affect children [124], while SS is a rare cancer type that affects soft tissues in children and adults [125]. Using both short hairpin RNA (shRNA) and dBRD9 chemical degradation, Michel et al. demonstrated that BRD9 suppression caused rapid attenuation of proliferation in these cancer types, once again identifying the potential utility for BRD9 as a therapeutic target [107]. A similar result was seen when evaluating the effects of two specific chemical probes targeting the BRD9 bromodomain in MRT cells [126]. The combined treatment of the bromodomain inhibitor I-BRD9 with certain cytotoxic drugs further attenuated the anti-proliferative effects seen by only I-BRD9 treatment, highlighting an avenue for a possible synergistic therapeutic strategy in MRT cases [126]. Additional research by Brien et al. showed that BRD9 degradation was particularly effective as a hindrance to SS viability [127], further implicating BRD9 as a critical component in BAF-perturbed cancers and a potential therapeutic target.

Of notable interest, Michel et al. also found a synthetic-lethal relationship between BRD9 and BAF in both MRT cells and SS. More specifically, when combined with BAF47-perturbed cells, depletion of BRD9 led to decreased proliferation in these cancer types [107]. Moreover, GBAF-specific functions such as promoter and CTCF site localization proceeded regardless of BAF complex perturbation, but the destabilization of GBAF by BRD9 degradation caused dysregulation of gene expression maintenance in both MRTs and SS [107]. Building upon this, special emphasis should be placed on the discovery by Michel et al. that loss of core BAF function in canonical BAF-perturbed cancers (e.g., loss of a core subunit such as BAF47) results in GBAF-dependent maintenance of gene expression to ensure cell survival. Due to the essential nature of GBAF under these conditions, the vulnerability of MRT and SS to BRD9 depletion is indirect; i.e., BAF47 deficiency makes these cancers vulnerable to BRD9 inhibition/depletion.

BRD9 is amplified across several other cancer types. A recent analysis of TCGA data from cBioPortal found that BRD9 was amplified in bladder cancer, lung squamous cell carcinoma, lung adenocarcinoma, esophageal carcinoma, and ovarian cancer, with several of these cancer types also displaying BAF53a amplification [128] (see Fig. 3). Antiproliferative synergy has also been observed in triple negative breast cancer when the bromodomains of BRD9 and bromodomain adjacent zinc finger BAZ2A are inhibited concurrently [129]. In addition, there appears to be a connection between splicing factor protein SF3B1 mutations and BRD9 degradation in cancers like myeloid leukemia, lymphoid leukemia, and uveal melanoma [130]. *SF3B1* is recurrently mutated in these cancers, and in fact, SF3B1 may be the most frequently mutated RNA splicing factor across all cancer types [131, 132]. Inoue et al. found that multiple *SF3B1* mutations converge upon BRD9 with repressive consequences, in an event caused by the inclusion of a poison exon in *BRD9* and subsequent mRNA degradation [130]. Furthermore, reduction in BRD9 levels lead to melanoma tumorigenesis, possibly due to decreased regulation of the *HTRA1* tumor suppressor caused by GBAF perturbation [130]. The authors also investigated the effects of antisense oligonucleotide (ASO) treatment as a way to prevent the poison exon inclusion. Correction of *BRD9* mis-splicing restored tumor suppressor activity of BRD9, as well as mRNA and protein levels, revealing a promising route for future research into *BRD9* mis-splicing-targeted therapies [130].

New research has also identified a link between GBAF and Merkel cell carcinoma MCC disease progression. Within this cancer type, the histone demethylase Lysine-specific histone demethylase 1 (LSD1) has a role in maintaining MCC growth under both in vitro and in vivo conditions [133]. Park et al. determined an antagonistic relationship exists between LSD1 and GBAF. Interestingly, inhibition of LSD1 resulted in decreased MCC cell viability, but this effect was rescued by BRD9 inhibition [133]. BRD9 was also found to be essential for LSD1 target gene expression, as BRD9 degradation negatively affected LSD1 target gene induction. These results provide a possible tumor suppressor role for GBAF in this cancer type, as well as a potential novel approach for targeted therapy.

Advances in RNA-targeted cancer therapies are being made which contribute to novel approaches for treating cancers affected by splicing factor mutations [134]. Strategies using ASOs to restore levels of affected proteins provide a compelling opportunity for future research in both cancer and other pathologies [135, 136]. Given the numerous disease implications involving BRD9, understanding the pathogenic contributions of this GBAF subunit merits continued study. Indeed, greater accessibility to selective BRD9 inhibitors will aid in further investigation of this subunit, creating new possibilities for teasing out the intricacies of SWI/SNF subunits in disease and development.

Once again, BAF subunits part of, but not exclusive to, GBAF are included in the following sections to facilitate conceptualization of GBAF subunit contributions to disease development as a whole. The information presented for these subunits is not exhaustive. Rather, it is given to underscore the complexities and the possible interactions and dependencies between GBAF and other BAF complexes.

### BRG1

BRG1 has known roles in regulating cellular proliferation in mammals [89, 137], and its purported tumor suppressor function is bolstered by the fact that *Brg1* is frequently mutated or deleted in murine tumor cell lines [138] and in approximately ten percent of human cancer cell lines [139]. BRG1 has been shown to be necessary for acute myeloid leukemia (AML) progression in both human cell lines and mouse models [140, 141]. Across small cell ovarian cancer types, BRG1 is mutated over 90% of the time [142, 143]. Heterozygous loss of *Brg1* in mice can lead to neoplasia and development of large subcutaneous tumors [60], indicating one function of BRG1 as a tumor suppressor.

Bai and colleagues showed that knockdown of BRG1 by RNAi greatly reduced breast cancer cell proliferation and induced cell cycle arrest [144]. BRG1 overexpression in human hepatocellular carcinoma samples further suggests that BRG1 can function in both tumor suppressing and oncogenic capacities depending upon oncogenic stimuli [145]. The potential therapeutic effects of targeting of BRG1 ATPase activity were recently assessed [146, 147].

### BAF155

Increased expression of BAF155 mRNA has been found in colorectal cancer, prostate cancer, and cervical intraepithelial neoplasia [148–151], and loss of BAF155 is seen in a variety of human cancers, including the A427 lung cancer cell line [152]. SRG3, a mouse homolog to BAF155, is a p53 target with critical roles in maintaining SWI/SNF complex stability and nuclear localization of several complex subunits [153–156]. Methylation of BAF155 appears to have a significant regulatory role in breast cancer cell migration and metastasis, as increased methylation of BAF155 mediated by coactivator-associated arginine methyltransferase 1 (CARM1) is associated with poor survival and metastatic lung colonization in mice [157]. More recently, links between BAF155 and tumor suppression in prostate cancer cell lines were uncovered, particularly with regards to regulation of tumor cell proliferation and migration [158].

### BAF53a

The human papillomavirus (HPV) oncogenes E6 and E7 function to counteract tumor suppressors like p53 and Rb, contributing to cervical cancer progression [159–161]. Knockdown of BAF53a in HeLa and SiHa cells has been shown to decrease expression of E6 and E7 genes, restoring the p53-dependent signaling pathways in these cell types [162]. Increased levels of BAF53a are seen in gliomas and may be an indicator of poor prognosis [163]. One of the most frequently mutated pathways in mammalian cancers involves the Myc TF family [163], and BAF53a is a known Myc cofactor critical for Myc-mediated oncogenesis in vivo [164].

BAF53a silencing has been explored as a target for attenuating rhabdomyosarcoma (RMS). Inhibition of this subunit resulted in embryonic RMS tumor suppression, prevention of proliferation and anchorage-independent growth, and induction of differentiation in RMS cell lines and mouse models [165].

### BAF60a

Nearly two decades ago, it was determined that BAF60a could interact with Fos/Jun heterodimers, with BAF60a serving as a determinant of the transactivation potential of these dimers and contributing to their roles in regulating cell growth, development, and differentiation [166]. Several members of both Fos and Jun gene families have been cloned and identified as homologs to viral oncogenes [167–169].

Recent work has demonstrated that multiple associations between BAF60a and microRNA (miRNA) targeting exist in carcinogenic pathways [170–172], providing a possible treatment approach as miRNA therapeutic strategies continue to evolve [reviewed by 173]. BAF60a is directly targeted by members of the tumor-suppressive mi-R99 family in prostate cancer [174], and mi-RNA-mediated post-transcriptional downregulation of BAF60a may play a role in controlling self-renewal in breast cancer-like stem cells [175]. BAF60a also directly interacts with p53 [176, 177], and it may aid in increasing efficacy of chemotherapy in some lung cancers [171].

### SS18

SS18 derives its name from a chromosomal translocation event that is frequently associated with tumorigenesis in synovial sarcoma [178]. The resulting translocation causes *SS18* (also known as *SSXT* or *SYT*) to fuse to either *SSX1*, *SSX2*, or *SSX4* from the X-chromosome (also known as *SS18-SSX*) [179–182]. The SS18 component of this fusion protein interacts with SWI/SNF and is primarily involved in transcriptional activation, whereas the SSX component interacts with the Polycomb complex and is mainly involved in transcriptional repression [183, 184]. Early studies into SS observed that despite loss of p53-mediated tumor suppression, the number of *p53* mutations was relatively low compared to other cancer types [185, 186]. This led researchers to discover that SS18-SSX negatively regulates p53 expression in SS by increasing the ubiquitination of p53 by HDM2, a negative regulator involved in a feedback loop controlling normal p53 expression [182].

The SS18-SSX fusion protein targets BAF complexes, and incorporation of this oncoprotein destabilizes BAF47 [187]. Knockdown of the SS18-SSX fusion protein in synovial sarcoma displayed similar anti-proliferative effects compared to treatment with the BRD9 chemical inhibitor dBRD9, but further investigation also uncovered that full degradation of BRD9 by this inhibitor did not affect SS18-SSX-mediated gene activation [107]. Furthermore, GBAF complexes appeared to preferentially associate with wild-type SS18 and were, in general, less affected by fusion oncoprotein perturbations. However, both MRT and synovial sarcoma maintain synthetic-lethal dependencies on GBAF. The authors postulate that one explanation for this is a collaboration between the compromised BAF complex and GBAF to account for the loss of BAF complex function, a conclusion strengthened by the fact that GBAF complex regulation primarily targets fusion-independent sites [107].

Currently, the SS18-SSX oncoprotein does not have viable drug prospects, but with more information and insight being generated regarding SS treatment, more novel therapies are being conceived and developed [reviewed by 188, 189–192]. Nonetheless, the limitations in our knowledge about synovial sarcoma progression, and resistance development in particular, solidify the need to further develop viable strategies to target this cancer type.

## Conclusions

Without a doubt, the complexities of SWI/SNF complex associations in different aspects of mammalian development and differentiation have resulted in a great challenge for researchers across multiple disciplines in the life sciences. While our knowledge base concerning these complexes is ever-expanding, it is still clear that many informational facets are yet unknown. Further insight into the molecular mechanisms at work in chromatin remodeler function carries the great potential of unlocking some of the mysteries and challenges being faced by researchers at present. GBAF subunits play an integral role in nearly every stage of life, from maintaining embryonic development, to regulating neural development and hematopoiesis, and to promoting (or preventing) cancer progression in childhood and adulthood. Additionally, a putative role for BRD9 in T-cell development further solidifies the need for closer examination of GBAF and its dedicated subunits within the contexts of pluripotency and immune system establishment. Though the mechanistic contributions of GBAF as a whole are still largely unknown, the significance of both its unique constituents and shared BAF subunits have in regulating mammalian development and disease provides compelling evidence for pursuing the contributions of GBAF in these areas.

GBAF subunit expression is seen across a large number of cancer types, and many of these subunits are embroiled in both tumor progression and suppression. Recent studies into the specific roles GBAF may have in cancer progression have underscored the significance of molecular and genetic interactions under these conditions. The ability of GBAF to maintain gene expression and cell survival in BAF47-deficient cancers serves as an intriguing opportunity for studies into therapeutic approaches targeting these cancers. Furthermore, new research identifying an antagonistic relationship between BRD9 and LDS1 provides a compelling basis for future exploration of GBAF tumor suppressor function.

Novel innovations are being developed to target pathologies associated with aberrant GBAF subunit expression and function. Advances in RNA-directed technologies are showing promise as potential therapeutic approaches in some cancer landscapes. RNAi, shRNA, and miRNA applications are being tested as possible ways to eliminate aberrant expression and restore tumor-suppressive activity in GBAF subunits with promising results. Moreover, recent studies implementing ASOs to target oncoproteins have shown promise, particularly in stubborn cancer types like MRT. Nonetheless, if anything is to be taken away from these studies, it is that therapeutic strategies must be applied in a context-specific manner, underpinning the truth that panacean approaches are rarely viable. Therefore, expanding our knowledge in all directions surrounding chromatin remodeler functions and interactions with other cellular components will be essential to unveil the intersections of these events and contribute to this expansion with greater impact.

## Data Availability

Not applicable.

## Abbreviations

AML: Acute myeloid leukemia
ASD: Autism spectrum disorder
ASO: Antisense oligonucleotide
BAF: BRM/BRG1-associated factors
BAP: Brahma-associated-proteins
BCL7: B-cell CLL/lymphoma 7 protein
BRD: Bromodomain
BRD4: Bromodomain-containing 4
BRD9: Bromodomain-containing 9
BRM: Brahma
CARM1: Coactivator-associated arginine methyltransferase 1
CRC: Colorectal cancer
Cryo-EM: Cryo-electron microscopy
CTCF: CCCTC-binding factor
EGFR: Epidermal growth factor receptor
esBAF: ESC BAF
ESCs: Embryonic stem cells
ESFT: Ewing’s sarcoma family tumor
GBAF: GLTSCR1/like-containing BAF complex
G-CSF: Granulocyte-colony stimulating factor
GLTSCR1: Glioma tumor suppressor candidate region gene 1
HPV: Human papillomavirus
ICM: Inner cell mass
miRNA: MicroRNA
MCC: Merkel cell carcinoma
MRTs: Malignant rhabdoid tumors
nBAF: Neuronal BAF
NCBRS: Nicolaides–Baraitser syndrome
npBAF: Neural progenitor BAF
PBAF: Polybromo-BAF
PRE: Primitive endoderm
PTM: Post-translational modification
RMS: Rhabdomyosarcoma
RNAi: RNA interference
shRNA: Short hairpin RNA
SNP: Single nucleotide polymorphism
SqCLC: Squamous cell lung cancer
SS: Synovial sarcoma
SWI/SNF: Switch/sucrose nonfermenting
TF: Transcription factor
Treg: Regulatory T-cell
ZGA: Zygotic genome activation
